# On the Performance of Energy Harvesting Dual-Hop Free-Space Optical Communication Systems with Secrecy Analysis

**DOI:** 10.3390/s25020319

**Published:** 2025-01-08

**Authors:** Abdulgani A. Ibrahim, Serdar Özgür Ata, Lütfiye Durak-Ata

**Affiliations:** 1 Informatics Institute, Istanbul Technical University, 34467 Istanbul, Turkey; durakata@itu.edu.tr; 2 Informatics and Information Security Research Center, TUBITAK, 41470 Kocaeli, Turkey; serdar.ata@tubitak.gov.tr

**Keywords:** dual hop, energy harvesting, FSO systems, performance analysis, physical layer security, SLIPT

## Abstract

In this study, we present a dual-hop decode-and-forward relaying-based free-space optical (FSO) communication system. We consider utilizing simultaneous lightwave information and power transfer (SLIPT) with a time-splitting technique at the relay, where the direct current component of the received optical signal is harvested as a transmit power for the relay. It is assumed that the FSO links experience a Malaga turbulence channel with pointing errors. In order to evaluate the performance of the proposed communication system, closed-form expressions for outage probability, ergodic capacity, average bit error rate, and throughput are derived. Additionally, to analyze the physical layer security of the proposed system, closed-form expressions for secrecy outage probability and strictly positive secrecy capacity are obtained. Finally, the accuracy of the derived analytical expressions are validated with Monte Carlo simulations. Results show that our proposed system model outperforms its non-SLIPT counterpart.

## 1. Introduction

Free-space optical (FSO) systems, a promising technology for the future sixth-generation (6G) wireless communication systems, possess attractive inherent advantages including ultra-high data rates, low cost, and ease of installation [1]. However, the performance of an FSO system is degraded due to its sensitivity to weather conditions, atmospheric turbulence-induced fading, and the effect of pointing errors, which is a result of the misalignment that occurs between the optical transmitter and the receiving aperture [2]. Statistical distributions, such as the Malaga distribution, have been developed to accurately model the effects of atmospheric turbulence and pointing errors [3].

The adverse effects of weather conditions, atmospheric turbulence, and pointing errors on the performance of FSO systems can be combated by deploying a relay between the transmitter and the receiver [4]. Relaying techniques also extend the transmission coverage and mitigate the short range issue of FSO systems [5]. Relaying can be implemented as FSO/FSO systems where all hops between the source and the destination are FSO links [6]. Additionally, mixed FSO/radio frequency (RF) systems can also be implemented [7] where one of the hops will operate as an RF link. Relay-assisted FSO systems are well studied in the literature (please refer to [8] and the references cited therein.). For instance, the study in [9] provides average bit error rate (BER), outage probability (OP), and ergodic capacity expressions of a dual-hop (DH) FSO/FSO fixed-gain relaying system over a Gamma–Gamma turbulence channel with pointing errors. Further, the work in [10] presents average BER and OP expressions of a multi-hop FSO system with an amplify-and-forward (AF) relaying technique over a double-generalized Gamma turbulence channel with pointing errors. Also, the study in [11] derives average BER, OP, and ergodic capacity expressions of a DH FSO/FSO system with a decode-and-forward (DF) relaying technique over a Malaga turbulence channel with pointing errors.

In relay-assisted FSO systems, the optical signal received at the relay is composed of an alternating current (AC) component that carries the information and a direct current (DC) component that guarantees the non-negativity of the optical signal [12]. Recently, the simultaneous lightwave information and power transfer (SLIPT) concept has been introduced, where the relay can harvest energy from the received optical signal by extracting its DC component and use it as a transmit power [13]. By adopting SLIPT, the received optical signal is not just utilized for information reception, but also for energy harvesting (EH) to wirelessly deliver energy to the relay. EH, which minimizes frequent battery replacements and material waste, plays a crucial role in fulfilling the sustainability goals of 6G networks [14]. Proof-of-concept experiments regarding optical energy harvesting have been presented in [15,16]. Studies regarding the performance of SLIPT-based FSO systems are still scarce in the literature [17]. In [18], OP expression of a SLIPT-based AF FSO/RF system is provided. Also, the work in [19] derives OP, average BER, and ergodic capacity expressions of a SLIPT-based mixed DF FSO/RF system. Furthermore, the study in [20] presents OP, coverage probability, and throughput expressions of a SLIPT-based FSO/RF system with a non-orthogonal multiple access technique. It should also be noted that SLIPT is proposed for indoor [21,22] and underwater [23,24] wireless optical communication systems.

Physical layer security [25] addresses the confidentiality of the wirelessly transmitted information without utilizing any encryption techniques in the upper layer of the network stack [26]. Although FSO systems are more secure than their RF counterparts [27], FSO links are also susceptible to eavesdropping due to the atmospheric turbulence, pointing errors, and the nature of the optical beam divergence [28]. Physical layer security for FSO communication systems is fairly studied in the literature [29]. For instance, secrecy outage probability (SOP) and average secrecy capacity (ASC) expressions of FSO links over F turbulence channels are provided in [30]. In [31], the SOP expression of an FSO system over a correlated Malaga turbulence channel is derived. Also, SOP and strictly positive secrecy capacity (SPSC) expressions of a mixed FSO/RF system are presented in [32]. Further, the secrecy performance of a parallel FSO/mm-wave system over an F turbulence channel is studied in [33].

It can be observed from the aforementioned literature work that the studies regarding SLIPT-based FSO communication systems are still limited and, to the best of the authors’ knowledge, SLIPT-based DH FSO/FSO communication systems are not studied in the open literature. Thus, in this work, we propose a DH DF-based FSO/FSO communication system where the relay harvests energy from the received optical signal through the SLIPT technique. We consider time-splitting [12]-based SLIPT where a portion of the transmission time is dedicated for energy harvesting. We also consider an eavesdropper at the relay to evaluate the physical layer security of the proposed communication system model. The main contributions of this study can be summarized as follows:

- We derive the probability distribution function (PDF) and the cumulative distribution function (CDF) expressions of the end-to-end (e2e) instantaneous signal-to-noise ratio (SNR) for the proposed SLIPT-based DH FSO communication system.
- By utilizing the derived CDF and PDF expressions, we obtain the OP, ergodic capacity, average BER, and throughput expressions of the proposed communication system.
- To analyze the physical layer security of the communication system, SOP and SPSC expressions are also presented.
- Finally, Monte Carlo simulations are carried out to validate the derived analytical expressions and to analyze different system parameters of the proposed communication system.

The rest of this paper is organized as follows: In Section 2, the system model, statistical characterization of the turbulence channels, and EH at the relay descriptions are provided. In Section 3, expressions such as OP, ergodic capacity, average BER, throughput, SOP, and SPSC are derived. Section 4 presents the simulations, and Section 5 concludes the study.

## 2. System and Channel Models

In this paper, we consider a DH FSO communication system with a relay that utilizes a DF-based relaying scheme. We also consider that the relay is harvesting energy from the received optical signal through SLIPT with a time-splitting technique. Both source-to-relay and relay-to-destination nodes use FSO communication links that undergo weather conditions (atmospheric attenuation), atmospheric turbulence, and pointing error effects. To explore the physical layer security of the proposed communication system, we also assume an eavesdropper that tries to intercept confidential information at the relay. We consider that the eavesdropper is located close to the legitimate relay in the divergence region of the laser beam, and that the eavesdropper can intercept enough optical power that can threaten the security of the transmitted information.

**Figure 1:**
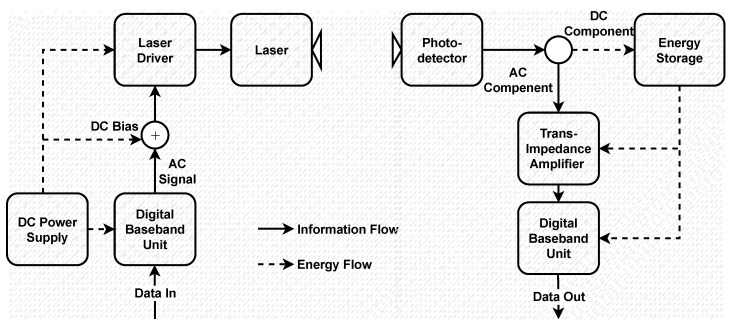
An illustration of a SLIPT transceiver architecture [13].

We assume that, as illustrated in Figure 1, the source node converts digitally modulated information signal, $X_{m}\left(t\right)$, to an optical signal. A DC bias $B\in \left(B_{\min},B_{\max}\right)$ is added to $X_{m}\left(t\right)$ to ensure a non-negative optical signal, where $B_{\min}$ and $B_{\max}$ are the minimum and maximum DC bias values, respectively. Let $P_{S}$ be the electrical power of the source node to transmit the optical signal S(t). Then, S(t) can be described as

(1) $$\begin{array}{c} S\left(t\right)=\sqrt{P_{S}}\left\{\zeta X_{m}\left(t\right)+B\right\}, \end{array}$$

where ζ is the electrical-to-optical conversion coefficient at the source node. To avoid clipping due to the nonlinear behavior of the laser, ζ is given as [34]

(2) $$\begin{array}{c} \zeta \leq \min\left(\frac{B-B_{\min}}{v},\frac{B_{\max}-B}{v}\right), \end{array}$$

where *v* is the power of $X_{m}\left(t\right)$. The electrical signal available at the output of the photo-detector (P-D) at the relay, $y_{R}\left(t\right)$, and at the eavesdropper, $y_{E}\left(t\right)$, can be described as

(3) $$\begin{array}{c} y_{i}\left(t\right)=\eta_{i}R_{i}A_{i}h_{i}S\left(t\right)+n_{i}\left(t\right),\;\;\;\;i\in \left\{R,E\right\}, \end{array}$$

where $\eta_{i}$ is the optical-to-electrical conversion coefficient. $R_{i}$ and $A_{i}$ are the responsivity and the detection area of the P-D, respectively. $h_{i}$ is the channel gain of the FSO link, and $n_{i}$ stands for a zero-mean additive white Gaussian noise (AWGN) with power $\sigma_{i}^{2}$. Noises at the P-D are the summation of many transmitted-signal-independent random variables. In the case of FSO systems with wide-field-of-view P-Ds, noises are adopted as AWGN with zero mean and variance $\sigma^{2}$. Please refer to [35] [Chapter 2] on more about the statistical modeling of the noises. Furthermore, the received signal at the destination node can be written as

(4) $$\begin{array}{c} y_{D}\left(t\right)=\sqrt{P_{R}}\eta_{D}R_{D}A_{D}h_{D}\zeta_{D}{\tilde{X}}_{m}\left(t\right)+n_{D}\left(t\right), \end{array}$$

where $P_{R}$ is the transmit power at the relay node, $\zeta_{D}$ is the electrical-to-optical conversion coefficient at the relay, ${\tilde{X}}_{m}$ is the information signal transmitted by the relay to the destination, and $n_{D}$ is a zero-mean AWGN with power $\sigma_{D}^{2}$.

### 2.1. Channel Model

We assume all FSO links experience Malaga-distributed atmospheric turbulence with the effect of pointing errors. The composite PDF of the Malaga fading channel with pointing errors and atmospheric attenuation effects is given as [2,36]

(5) $$\begin{array}{c} f_{h}\left(h\right)=\frac{\xi^{2}A}{2h}\sum_{k=1}^{\beta }b_{k}G_{1,3}^{3,0}\left(\frac{\alpha \beta h}{h_{l}\left(g\beta +\Omega^{\prime }\right)A_{0}}|\begin{array}{c} \xi^{2}+1\\ \xi^{2},\alpha ,k \end{array}\right), \end{array}$$

where

(6) A=2αα2g1+α2Γαgβgβ+Ω′β+α2,bk=β−1k−1αk2gβ+Ω′1−k2Ω′k−1βk2gk−1k−1!αβgβ+Ω′−α+k2.

In (5) and (6), α and β are atmospheric fading parameters which correspond to the large-scale and small-scale atmospheric fluctuations, respectively. $g=2b_{0}\left(1-p\right)$ and $\Omega^{\prime }=\Omega +p2b_{0}+2\sqrt{2b_{0}\Omega p}\cos\left(\Phi_{A}-\Phi_{B}\right)$. Here, Ω is the average power of the optical signal for the line-of-sight (LOS) component, $2b_{0}$ is the average power of total scatter components, and *p* is the amount of the scattering power coupled to the LOS component. Further, $\Phi_{A}$ and $\Phi_{B}$ are deterministic angles for the LOS component and the coupled-to-LOS scatter component, respectively. $h_{l}$ is the atmospheric attenuation due to the weather conditions which can be calculated, by using the Beer–Lambert law, as $h_{l}=\exp\left(-\omega L\right)$, where ω is the atmospheric attenuation coefficient and *L* is the FSO link distance. In addition, $\xi =\frac{w_{zeq}}{2\sigma_{s}}$ defines the ratio between the equivalent beam radius at the receiver and the pointing error displacement standard deviation at the receiver. Also, $\sigma_{s}^{2}$ is the jitter variance at the receiver and $A_{0}={\left(\mathrm{erf}\left(v\right)\right)}^{2}$ is the fraction of the collected power with zero pointing errors. $v=\frac{\sqrt{\pi }a}{\sqrt{2}w_{z}}$ and $w_{zeq}^{2}=w_{z}^{2}\frac{\sqrt{\pi }\mathrm{erf}\left(v\right)}{2v\exp(-v^{2})}$. And $w_{z}$ is the beam waist at distance *z* and $w_{zeq}$ is the equivalent beam waist.

### 2.2. Energy Harvesting at the Relay

**Figure 2:**
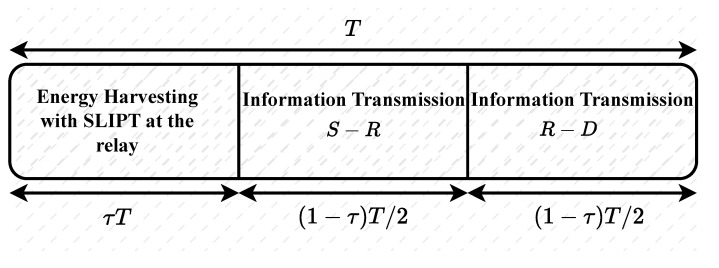
An illustration of the SLIPT-based time-splitting technique.

The received optical signal is converted into an electrical signal at the P-D. Then, this electrical signal is separated into AC and DC components. We consider collecting the DC component from (3), $\eta_{R}R_{R}A_{R}h_{R}\sqrt{P_{S}}B$, which is generally filtered out, as the transmit power for the relay. Meanwhile, the AC component, which contains the information signal, is modulated and re-transmitted to the destination. By applying the time-splitting technique, as illustrated in Figure 2, a portion of the available block transmission time is dedicated for energy harvesting at the relay, and the other portion is utilized for information transmission.

Let *T* (in seconds) denote the block transmission time and τ be the portion of the time that is solely allocated for the phase in which only energy is harvested at the relay. During this phase of EH, the transmitter eliminates the AC component and maximizes the DC bias, i.e., ζ=0 and $B=B_{\max}$ in (1). The amount of energy harvested during this first phase is given as

(7) $$\begin{array}{c} E_{h}^{\left(1\right)}=\frac{0.75V_{t}{\left(\eta_{R}R_{R}A_{R}h_{R}\sqrt{P_{S}}B_{\max}\right)}^{2}}{I_{0}}\left(\tau T\right), \end{array}$$

where $V_{t}$ and $I_{0}$ are the thermal voltage and the dark saturation current of the P-D, respectively [34]. In the second phase, the objective is to maximize the received SNR at the relay. In this second phase, both the AC component for information decoding and the inherent DC component of the optical signal are transmitted. To maximize the SNR, we set $\zeta =\frac{B_{\max}-B_{\min}}{2v}$ and $B=\frac{B_{\max}+B_{\min}}{2}$ in (1). Thus, the energy harvested in this second phase is given as

(8) $$\begin{array}{c} E_{h}^{\left(2\right)}=\frac{0.75V_{t}{\left(\eta_{R}R_{R}A_{R}h_{R}\sqrt{P_{S}}\left(\frac{B_{\max}+B_{\min}}{2}\right)\right)}^{2}}{I_{0}}\frac{\left(1-\tau \right)T}{2}. \end{array}$$

The total harvested energy for one block transmission time is, therefore, given as

(9) $$\begin{array}{c} E_{h}=E_{h}^{\left(1\right)}+E_{h}^{\left(2\right)}, \end{array}$$

and the harvested power available at the relay is given as

(10) $$\begin{array}{cc} P_{R} & =\frac{E_{h}}{\left(1-\tau )(T/2\right)}=\frac{0.75V_{t}{\left(\eta_{R}R_{R}A_{R}h_{R}\sqrt{P_{S}}\right)}^{2}}{I_{0}}\left(\frac{2\tau B_{\max}^{2}}{\left(1-\tau \right)}+{\left(\frac{B_{\max}+B_{\min}}{2}\right)}^{2}\right). \end{array}$$

### 2.3. Instantaneous SNR Characterization

The instantaneous SNR at the relay and eavesdropper can be written from (3) as $\gamma_{i}=\mu_{i}h_{i}^{2}$, where $i\in \left\{R,E\right\}$ and $\mu_{i}={\left(\eta_{i}R_{i}A_{i}\sqrt{P_{S}}\zeta \right)}^{2}E^{2}\left[h_{i}\right]/\sigma_{i}^{2}$ is the average SNR. It is assumed that the FSO links’ channel gains, $h_{i}$, follow the Malaga distribution with pointing errors, and the PDF and CDF expressions of $\gamma_{i}$ can be calculated from (5) as [36]

(11) $$\begin{array}{c} f_{\gamma_{i}}\left(x\right)=\sum_{k=1}^{\beta_{i}}\frac{\phi_{1i}}{x}G_{1,3}^{3,0}\left(\frac{\psi_{1i}x^{\frac{1}{2}}}{\mu_{i}^{\frac{1}{2}}}|\begin{array}{c} \xi_{i}^{2}+1\\ \xi_{i}^{2},\alpha_{i},k \end{array}\right),\;\;\;\;\;i\in \left\{R,E\right\}; \end{array}$$

(12) $$\begin{array}{c} F_{\gamma_{i}}\left(x\right)=\sum_{k=1}^{\beta_{i}}\phi_{2i}G_{3,7}^{6,1}\left(\frac{\psi_{1i}^{2}x}{2^{4}\mu_{i}}|\begin{array}{c} 1,\kappa_{1i}\\ \kappa_{2i},0 \end{array}\right),\;\;\;\;\;i\in \left\{R,E\right\}, \end{array}$$

where $\kappa_{1i}=\Delta (2:\;1+\xi_{i}^{2})$, $\kappa_{2i}=\Delta (2:\;\xi_{i}^{2}),\;\Delta (2:\;\alpha_{i}),\;\Delta (2:\;k)$, $\phi_{1i}=\frac{\xi_{i}^{2}A_{i}b_{k}}{4}$, $\psi_{1i}=\frac{\xi_{i}^{2}\alpha_{i}\beta_{i}\left(g_{i}+\Omega_{i}^{\prime }\right)}{\left(g_{i}\beta_{i}+\Omega_{i}^{\prime }\right)\left(1+\xi_{i}^{2}\right)}$, and $\phi_{2i}=\frac{\xi_{i}^{2}A_{i}b_{k}2^{\alpha_{i}+k-4}}{\pi }$. $A_{i}$ and $b_{k}$ are given in (6). Here, $\Delta \left(p:q\right)=\frac{q}{p},\ldots ,\frac{q+p-1}{p}$ is a notation for a vector comprising *p* terms.

The instantaneous SNR at the destination node can be calculated from (4) as $\gamma_{D}=\mu_{D}h_{D}^{2}$, where $\mu_{D}={\left(\eta_{D}R_{D}A_{D}\sqrt{P_{R}}\zeta_{D}\right)}^{2}E^{2}\left[h_{D}\right]/\sigma_{D}^{2}$. Additionally, as a DF relaying scheme is assumed at the relay node, the e2e instantaneous SNR for the communication link can be described as

(13) $$\begin{array}{c} \gamma_{e2e}=\min\left(\gamma_{R},\gamma_{D}\right). \end{array}$$

## 3. Performance Analysis

In this section, we present OP, ergodic capacity, average BER, throughput, SOP, and SPSC expressions for the proposed communication system model.

### 3.1. Outage Probability

The CDF of e2e instantaneous SNR can be calculated from (13) as

(14) $$\begin{array}{c} F_{\gamma_{e2e}}\left(z\right)=\mathrm{Pr}\left[\min\left(\gamma_{R},\gamma_{D}\right)\leq z\right]. \end{array}$$

It can be observed that $\gamma_{R}$ and $\gamma_{D}$ in (14) are two dependent random variables (RVs), since the $P_{R}$ in (10), which is the harvested energy at the relay, depends on the channel gain $h_{R}$.

**Lemma** **1.** *The CDF of $\gamma_{e2e}$ is given as*

(15) $$\begin{array}{cc} F_{\gamma_{e2e}}\left(z\right) & =\sum_{k=1}^{\beta_{R}}\sum_{l=1}^{\beta_{D}}\frac{\Phi_{1}\phi_{2R}}{\mu_{0}^{\varrho_{1}}}G_{4,8}^{7,1}\left(\frac{\psi_{1R}^{2}z}{2^{4}\mu_{R}}|\begin{array}{c} 1,\kappa_{1R},1+\varrho_{1}\\ \varrho_{1},\kappa_{2R},0 \end{array}\right)+\sum_{k=1}^{\beta_{R}}\sum_{l=1}^{\beta_{D}}\sum_{n=0}^{N}\frac{\Phi_{2}\phi_{2R}}{\mu_{0}^{\varrho_{2}}}G_{4,8}^{7,1}\left(\frac{\psi_{1R}^{2}z}{2^{4}\mu_{R}}|\begin{array}{c} 1,\kappa_{1R},1+\varrho_{2}\\ \varrho_{2},\kappa_{2R},0 \end{array}\right)\\ \; & +\sum_{k=1}^{\beta_{R}}\sum_{l=1}^{\beta_{D}}\sum_{n=0}^{N}\frac{\Phi_{3}\phi_{2R}}{\mu_{0}^{\varrho_{3}}}G_{4,8}^{7,1}\left(\frac{\psi_{1R}^{2}z}{2^{4}\mu_{R}}|\begin{array}{c} 1,\kappa_{1R},1+\varrho_{3}\\ \varrho_{3},\kappa_{2R},0 \end{array}\right)+\sum_{k=1}^{\beta_{R}}\left(1-F_{\gamma_{D}^{\prime }}\left(1\right)\right)\phi_{2R}G_{3,7}^{6,1}\left(\frac{\psi_{1R}^{2}z}{2^{4}\mu_{R}}|\begin{array}{c} 1,\kappa_{1R}\\ \kappa_{2R},0 \end{array}\right), \end{array}$$

*where $\mu_{0}$ is given in (A1), $\varrho_{1}=\frac{\xi_{D}^{2}}{2}$, $\varrho_{2}=\frac{n+\alpha_{D}}{2}$, and $\varrho_{3}=\frac{n+l}{2}$. Also, $\Phi_{1}$, $\Phi_{2}$, and $\Phi_{3}$ are given in (A7). Further, $F_{\gamma_{D}^{\prime }}\left(1\right)$ can be obtained from (A5).*

**Proof.**  Please refer to the Appendix A for the proof of (15). □

The outage probability of the communication system is defined as the probability that the received e2e instantaneous SNR is smaller than a predefined threshold, $\gamma_{\mathrm{th}}$, and it can be expressed as

(16) $$\begin{array}{c} P_{\mathrm{out}}=\mathrm{Pr}\left[\gamma_{e2e}\leq \gamma_{\mathrm{th}}\right]=F_{\gamma_{e2e}}\left(\gamma_{\mathrm{th}}\right), \end{array}$$

where $F_{\gamma_{e2e}}\left(\gamma_{\mathrm{th}}\right)$ can be obtained from (15).

### 3.2. Ergodic Capacity

The ergodic capacity of intensity-modulated FSO systems can be calculated based on [37] [Equation (25)] and [38] [Equation (42)]:

(17) $$\begin{array}{c} \bar{C}=\frac{1}{\log\left(2\right)}\int_{0}^{\infty }\log\left(1+\lambda \gamma \right)f_{\gamma_{e2e}}\left(\gamma \right)d\gamma , \end{array}$$

where $\lambda =\frac{\exp(1)}{2\pi }$ is a constant term for intensity modulation/direct detection technique-based FSO systems [39], and $f_{\gamma_{e2e}}\left(\gamma \right)$ is the PDF expression for the e2e instantaneous SNR.

**Lemma** **2.** *The PDF of $\gamma_{e2e}$ is calculated as*

(18) $$\begin{array}{cc} f_{\gamma_{e2e}}\left(z\right) & =\sum_{k=1}^{\beta_{R}}\sum_{l=1}^{\beta_{D}}\frac{\Phi_{1}\phi_{2R}}{\mu_{0}^{\varrho_{1}}}z^{-1}G_{3,7}^{7,0}\left(\frac{\psi_{1R}^{2}z}{2^{4}\mu_{R}}|\begin{array}{c} \kappa_{1R},1+\varrho_{1}\\ \varrho_{1},\kappa_{2R} \end{array}\right)+\sum_{k=1}^{\beta_{R}}\sum_{l=1}^{\beta_{D}}\sum_{n=0}^{N}\frac{\Phi_{2}\phi_{2R}}{\mu_{0}^{\varrho_{2}}}z^{-1}G_{3,7}^{7,0}\left(\frac{\psi_{1R}^{2}z}{2^{4}\mu_{R}}|\begin{array}{c} \kappa_{1R},1+\varrho_{2}\\ \varrho_{2},\kappa_{2R} \end{array}\right)\\ \; & +\sum_{k=1}^{\beta_{R}}\sum_{l=1}^{\beta_{D}}\sum_{n=0}^{N}\frac{\Phi_{3}\phi_{2R}}{\mu_{0}^{\varrho_{3}}}z^{-1}G_{3,7}^{7,0}\left(\frac{\psi_{1R}^{2}z}{2^{4}\mu_{R}}|\begin{array}{c} \kappa_{1R},1+\varrho_{3}\\ \varrho_{3},\kappa_{2R} \end{array}\right)+\sum_{k=1}^{\beta_{R}}\left(1-F_{\gamma_{D}^{\prime }}\left(1\right)\right)\phi_{2R}z^{-1}G_{2,6}^{6,0}\left(\frac{\psi_{1R}^{2}z}{2^{4}\mu_{R}}|\begin{array}{c} \kappa_{1R}\\ \kappa_{2R} \end{array}\right). \end{array}$$

**Proof.**  The PDF of e2e SNR, $f_{\gamma_{e2e}}\left(z\right)$, is derived by differentiating (15), using [40] [Equation (07.34.20.0017.02)], as given in (18). □

Now, rewriting the log function in (17) as $\log\left(1+\lambda \gamma \right)=G_{2,2}^{1,2}\left(\lambda \gamma |\begin{array}{c} 1,1\\ 1,0 \end{array}\right)$ and using (18) in (17), we can write

(19) $$\begin{array}{cc} \; & \bar{C}=\sum_{k=1}^{\beta_{R}}\sum_{l=1}^{\beta_{D}}\frac{\Phi_{1}\phi_{2R}}{\log\left(2\right)\mu_{0}^{\varrho_{1}}}I_{1}+\sum_{k=1}^{\beta_{R}}\sum_{l=1}^{\beta_{D}}\sum_{n=0}^{N}\frac{\Phi_{2}\phi_{2R}}{\log\left(2\right)\mu_{0}^{\varrho_{2}}}I_{2}+\sum_{k=1}^{\beta_{R}}\sum_{l=1}^{\beta_{D}}\sum_{n=0}^{N}\frac{\Phi_{3}\phi_{2R}}{\log\left(2\right)\mu_{0}^{\varrho_{3}}}I_{3}+\sum_{k=1}^{\beta_{R}}\left(1-F_{\gamma_{D}^{\prime }}\left(1\right)\right)\frac{\phi_{2R}}{\log\left(2\right)}I_{4}, \end{array}$$

where

(20) $$\begin{array}{cc} \; & I_{1}=\int_{0}^{\infty }\gamma^{-1}G_{2,2}^{1,2}\left(\lambda \gamma |\begin{array}{c} 1,1\\ 1,0 \end{array}\right)G_{3,7}^{7,0}\left(\frac{\psi_{1R}^{2}\gamma }{2^{4}\mu_{R}}|\begin{array}{c} \kappa_{1R},1+\varrho_{1}\\ \varrho_{1},\kappa_{2R} \end{array}\right)d\gamma ;\\ \; & I_{2}=\int_{0}^{\infty }\gamma^{-1}G_{2,2}^{1,2}\left(\lambda \gamma |\begin{array}{c} 1,1\\ 1,0 \end{array}\right)G_{3,7}^{7,0}\left(\frac{\psi_{1R}^{2}\gamma }{2^{4}\mu_{R}}|\begin{array}{c} \kappa_{1R},1+\varrho_{2}\\ \varrho_{2},\kappa_{2R} \end{array}\right)d\gamma ;\\ \; & I_{3}=\int_{0}^{\infty }\gamma^{-1}G_{2,2}^{1,2}\left(\lambda \gamma |\begin{array}{c} 1,1\\ 1,0 \end{array}\right)G_{3,7}^{7,0}\left(\frac{\psi_{1R}^{2}\gamma }{2^{4}\mu_{R}}|\begin{array}{c} \kappa_{1R},1+\varrho_{3}\\ \varrho_{3},\kappa_{2R} \end{array}\right)d\gamma ;\\ \; & I_{4}=\int_{0}^{\infty }\gamma^{-1}G_{2,2}^{1,2}\left(\lambda \gamma |\begin{array}{c} 1,1\\ 1,0 \end{array}\right)G_{2,6}^{6,0}\left(\frac{\psi_{1R}^{2}\gamma }{2^{4}\mu_{R}}|\begin{array}{c} \kappa_{1R}\\ \kappa_{2R} \end{array}\right)d\gamma . \end{array}$$

The integrals in (20) can be solved by using [40] [Equation (07.34.21.0011.01)] as

(21) $$\begin{array}{cc} \; & I_{1}=G_{5,9}^{9,1}\left(\frac{\psi_{1R}^{2}}{\lambda 2^{4}\mu_{R}}|\begin{array}{c} 0,1,\kappa_{1R},1+\varrho_{1}\\ \varrho_{1},\kappa_{2R},0,0 \end{array}\right);\\ \; & I_{2}=G_{5,9}^{9,1}\left(\frac{\psi_{1R}^{2}}{\lambda 2^{4}\mu_{R}}|\begin{array}{c} 0,1,\kappa_{1R},1+\varrho_{2}\\ \varrho_{2},\kappa_{2R},0,0 \end{array}\right);\\ \; & I_{3}=G_{5,9}^{9,1}\left(\frac{\psi_{1R}^{2}}{\lambda 2^{4}\mu_{R}}|\begin{array}{c} 0,1,\kappa_{1R},1+\varrho_{3}\\ \varrho_{3},\kappa_{2R},0,0 \end{array}\right);\\ \; & I_{4}=G_{4,8}^{8,1}\left(\frac{\psi_{1R}^{2}}{\lambda 2^{4}\mu_{R}}|\begin{array}{c} 0,1,\kappa_{1R}\\ \kappa_{2R},0,0 \end{array}\right). \end{array}$$

The ergodic capacity expression is, therefore, obtained by using (21) in (19).

### 3.3. Average Bit Error Rate

The average BER expression of FSO communication systems with a binary phase shift keying modulation scheme can be calculated based on [19] [Equation (42)] and [11] [Equation (21)]:

(22) $$\begin{array}{c} P_{e}=\frac{1}{2\sqrt{\pi }}\int_{0}^{\infty }\gamma^{-\frac{1}{2}}\exp\left(-\gamma \right)F_{\gamma_{e2e}}\left(\gamma \right)d\gamma , \end{array}$$

where $F_{\gamma_{e2e}}\left(\gamma \right)$ is the CDF expression of the e2e SNR given in (15). Now, rewriting the exp function in (22) as $\exp\left(-\gamma \right)=G_{0,1}^{1,0}\left(\gamma |\begin{array}{c} -\\ 0 \end{array}\right)$ and using (15) in (22), we may write

(23) $$\begin{array}{c} P_{e}=\sum_{k=1}^{\beta_{R}}\sum_{l=1}^{\beta_{D}}\frac{\Phi_{1}\phi_{2R}}{2\sqrt{\pi }\mu_{0}^{\varrho_{1}}}I_{5}+\sum_{k=1}^{\beta_{R}}\sum_{l=1}^{\beta_{D}}\sum_{n=0}^{N}\frac{\Phi_{2}\phi_{2R}}{2\sqrt{\pi }\mu_{0}^{\varrho_{2}}}I_{6}+\sum_{k=1}^{\beta_{R}}\sum_{l=1}^{\beta_{D}}\sum_{n=0}^{N}\frac{\Phi_{3}\phi_{2R}}{2\sqrt{\pi }\mu_{0}^{\varrho_{3}}}I_{7}+\sum_{k=1}^{\beta_{R}}\left(1-F_{\gamma_{D}^{\prime }}\left(1\right)\right)\frac{\phi_{2R}}{2\sqrt{\pi }}I_{8}, \end{array}$$

where

(24) $$\begin{array}{cc} \; & I_{5}=\int_{0}^{\infty }\gamma^{-\frac{1}{2}}G_{0,1}^{1,0}\left(\gamma |\begin{array}{c} -\\ 0 \end{array}\right)G_{4,8}^{7,1}\left(\frac{\psi_{1R}^{2}\gamma }{2^{4}\mu_{R}}|\begin{array}{c} 1,\kappa_{1R},1+\varrho_{1}\\ \varrho_{1},\kappa_{2R},0 \end{array}\right)d\gamma ;\\ \; & I_{6}=\int_{0}^{\infty }\gamma^{-\frac{1}{2}}G_{0,1}^{1,0}\left(\gamma |\begin{array}{c} -\\ 0 \end{array}\right)G_{4,8}^{7,1}\left(\frac{\psi_{1R}^{2}\gamma }{2^{4}\mu_{R}}|\begin{array}{c} 1,\kappa_{1R},1+\varrho_{2}\\ \varrho_{2},\kappa_{2R},0 \end{array}\right)d\gamma ;\\ \; & I_{7}=\int_{0}^{\infty }\gamma^{-\frac{1}{2}}G_{0,1}^{1,0}\left(\gamma |\begin{array}{c} -\\ 0 \end{array}\right)G_{4,8}^{7,1}\left(\frac{\psi_{1R}^{2}\gamma }{2^{4}\mu_{R}}|\begin{array}{c} 1,\kappa_{1R},1+\varrho_{3}\\ \varrho_{3},\kappa_{2R},0 \end{array}\right)d\gamma ;\\ \; & I_{8}=\int_{0}^{\infty }\gamma^{-\frac{1}{2}}G_{0,1}^{1,0}\left(\gamma |\begin{array}{c} -\\ 0 \end{array}\right)G_{3,7}^{6,1}\left(\frac{\psi_{1R}^{2}\gamma }{2^{4}\mu_{R}}|\begin{array}{c} 1,\kappa_{1R}\\ \kappa_{2R},0 \end{array}\right)d\gamma . \end{array}$$

The integrals in (24) are solved with the help of [40] [Equation (07.34.21.0011.01)] as

(25) $$\begin{array}{cc} \; & I_{5}=G_{5,8}^{7,2}\left(\frac{\psi_{1R}^{2}}{2^{4}\mu_{R}}|\begin{array}{c} 1,\frac{1}{2},\kappa_{1R},1+\varrho_{1}\\ \varrho_{1},\kappa_{2R},0 \end{array}\right);\\ \; & I_{6}=G_{5,8}^{7,2}\left(\frac{\psi_{1R}^{2}}{2^{4}\mu_{R}}|\begin{array}{c} 1,\frac{1}{2},\kappa_{1R},1+\varrho_{2}\\ \varrho_{2},\kappa_{2R},0 \end{array}\right);\\ \; & I_{7}=G_{5,8}^{7,2}\left(\frac{\psi_{1R}^{2}}{2^{4}\mu_{R}}|\begin{array}{c} 1,\frac{1}{2},\kappa_{1R},1+\varrho_{3}\\ \varrho_{3},\kappa_{2R},0 \end{array}\right);\\ \; & I_{8}=G_{4,7}^{6,2}\left(\frac{\psi_{1R}^{2}}{2^{4}\mu_{R}}|\begin{array}{c} 1,\frac{1}{2},\kappa_{1R}\\ \kappa_{2R},0 \end{array}\right). \end{array}$$

The average BER expression is, thus, derived by plugging (25) into (23).

### 3.4. Throughput

The throughput, which is related to the effective information transmission time of the system, can be calculated as [41]

(26) $$\begin{array}{c} \mathrm{Throughput}=\frac{(1-\tau )T/2}{T}\bar{C}=\frac{\left(1-\tau \right)}{2}\bar{C}, \end{array}$$

where (1−τ)T/2 is the effective information transmission time between the source and the destination for the proposed communication system model.

### 3.5. Secrecy Outage Probability

SOP is defined as the probability that the instantaneous secrecy capacity, $C_{s}$, falls below a predetermined secrecy rate, $R_{s}\geq 0$. The secrecy capacity can be written as [32] [Equation (41)]

(27) $$\begin{array}{c} C_{s}\left(\gamma_{e2e},\gamma_{E}\right)=\max\left\{\log_{2}\left(1+\lambda \gamma_{e2e}\right)-\log_{2}\left(1+\lambda \gamma_{E}\right),0\right\}, \end{array}$$

where $\log_{2}\left(1+\lambda \gamma_{e2e}\right)$ and $\log_{2}\left(1+\lambda \gamma_{E}\right)$ denote the capacities of the legitimate receiver at the destination and the eavesdropper at the relay, respectively. SOP can be mathematically described as

(28) $$\begin{array}{cc} \mathrm{SOP} & =\mathrm{Pr}\left[C_{s}\left(\gamma_{e2e},\gamma_{E}\right)\leq R_{s}\right]=\int_{0}^{\infty }F_{\gamma_{e2e}}\left(\theta \gamma +\theta \lambda^{-1}-\lambda^{-1}\right)f_{\gamma_{E}}\left(\gamma \right)d\gamma , \end{array}$$

where $\theta =2^{R_{s}}$, $f_{\gamma_{E}}\left(\gamma \right)$ is the PDF of eavesdropper’s instantaneous SNR which is given in (11), and $F_{\gamma_{e2e}}\left(z\right)$ is the CDF of $\gamma_{e2e}$ which is given in (15). Finding the closed-form solution of the integral in (28) is challenging [42] [Equation (7)]. However, the lower bound of SOP (the case when γ→∞) [30] [Equation (18)] can be calculated as

(29) $$\begin{array}{c} {\mathrm{SOP}}_{L}=P\left\{\gamma_{e2e}\leq \theta \gamma_{E}\right\}=\int_{0}^{\infty }F_{\gamma_{e2e}}\left(\theta \gamma \right)f_{\gamma_{E}}\left(\gamma \right)d\gamma . \end{array}$$

Now, using (15) and (11) in (29), we can write

(30) $$\begin{array}{cc} {\mathrm{SOP}}_{L} & =\sum_{k=1}^{\beta_{R}}\sum_{l=1}^{\beta_{D}}\sum_{m=1}^{\beta_{E}}\frac{\Phi_{1}\phi_{2R}\phi_{1E}}{\mu_{0}^{\varrho_{1}}}I_{9}+\sum_{k=1}^{\beta_{R}}\sum_{l=1}^{\beta_{D}}\sum_{m=1}^{\beta_{E}}\sum_{n=0}^{N}\Phi_{2}\frac{\phi_{2R}\phi_{1E}}{\mu_{0}^{\varrho_{2}}}I_{10}\\ \; & +\sum_{k=1}^{\beta_{R}}\sum_{l=1}^{\beta_{D}}\sum_{m=1}^{\beta_{E}}\sum_{n=0}^{N}\frac{\Phi_{3}\phi_{2R}\phi_{1E}}{\mu_{0}^{\varrho_{3}}}I_{11}+\sum_{k=1}^{\beta_{R}}\sum_{m=1}^{\beta_{E}}\left(1-F_{\gamma_{D}^{\prime }}\left(1\right)\right)\phi_{2R}\phi_{1E}I_{12}, \end{array}$$

where

(31) $$\begin{array}{cc} \; & I_{9}=\int_{0}^{\infty }\gamma^{-1}G_{4,8}^{7,1}\left(\frac{\theta \psi_{1R}^{2}\gamma }{2^{4}\mu_{R}}|\begin{array}{c} 1,\kappa_{1R},1+\varrho_{1}\\ \varrho_{1},\kappa_{2R},0 \end{array}\right)G_{1,3}^{3,0}\left(\frac{\psi_{1E}\gamma^{\frac{1}{2}}}{\mu_{E}^{\frac{1}{2}}}|\begin{array}{c} \xi_{E}^{2}+1\\ \xi_{E}^{2},\alpha_{E},m \end{array}\right)d\gamma ;\\ \; & I_{10}=\int_{0}^{\infty }\gamma^{-1}G_{4,8}^{7,1}\left(\frac{\theta \psi_{1R}^{2}\gamma }{2^{4}\mu_{R}}|\begin{array}{c} 1,\kappa_{1R},1+\varrho_{2}\\ \varrho_{2},\kappa_{2R},0 \end{array}\right)G_{1,3}^{3,0}\left(\frac{\psi_{1E}\gamma^{\frac{1}{2}}}{\mu_{E}^{\frac{1}{2}}}|\begin{array}{c} \xi_{E}^{2}+1\\ \xi_{E}^{2},\alpha_{E},m \end{array}\right)d\gamma ,\\ \; & I_{11}=\int_{0}^{\infty }\gamma^{-1}G_{4,8}^{7,1}\left(\frac{\theta \psi_{1R}^{2}\gamma }{2^{4}\mu_{R}}|\begin{array}{c} 1,\kappa_{1R},1+\varrho_{3}\\ \varrho_{3},\kappa_{2R},0 \end{array}\right)G_{1,3}^{3,0}\left(\frac{\psi_{1E}\gamma^{\frac{1}{2}}}{\mu_{E}^{\frac{1}{2}}}|\begin{array}{c} \xi_{E}^{2}+1\\ \xi_{E}^{2},\alpha_{E},m \end{array}\right)d\gamma ;\\ \; & I_{12}=\int_{0}^{\infty }\gamma^{-1}G_{3,7}^{6,1}\left(\frac{\theta \psi_{1R}^{2}\gamma }{2^{4}\mu_{R}}|\begin{array}{c} 1,\kappa_{1R}\\ \kappa_{2R},0 \end{array}\right)G_{1,3}^{3,0}\left(\frac{\psi_{1E}\gamma^{\frac{1}{2}}}{\mu_{E}^{\frac{1}{2}}}|\begin{array}{c} \xi_{E}^{2}+1\\ \xi_{E}^{2},\alpha_{E},m \end{array}\right)d\gamma . \end{array}$$

The integrals in (31) are solved by employing [40] [Equation (07.34.21.0013.01)] as

(32) $$\begin{array}{cc} \; & I_{9}=\frac{2^{\alpha_{E}+m-2}}{\pi }G_{10,10}^{7,7}\left(\frac{\psi_{1E}^{2}\mu_{R}}{\theta \psi_{1R}^{2}\mu_{E}}|\begin{array}{c} 1-\varrho_{1},1-\kappa_{2R},1,\kappa_{1E}\\ \kappa_{2E},0,1-\kappa_{1R},-\varrho_{1} \end{array}\right);\\ \; & I_{10}=\frac{2^{\alpha_{E}+m-2}}{\pi }G_{10,10}^{7,7}\left(\frac{\psi_{1E}^{2}\mu_{R}}{\theta \psi_{1R}^{2}\mu_{E}}|\begin{array}{c} 1-\varrho_{2},1-\kappa_{2R},1,\kappa_{1E}\\ \kappa_{2E},0,1-\kappa_{1R},-\varrho_{2} \end{array}\right);\\ \; & I_{11}=\frac{2^{\alpha_{E}+m-2}}{\pi }G_{10,10}^{7,7}\left(\frac{\psi_{1E}^{2}\mu_{R}}{\theta \psi_{1R}^{2}\mu_{E}}|\begin{array}{c} 1-\varrho_{3},1-\kappa_{2R},1,\kappa_{1E}\\ \kappa_{2E},0,1-\kappa_{1R},-\varrho_{3} \end{array}\right);\\ \; & I_{12}=\frac{2^{\alpha_{E}+m-2}}{\pi }G_{9,9}^{7,6}\left(\frac{\psi_{1E}^{2}\mu_{R}}{\theta \psi_{1R}^{2}\mu_{E}}|\begin{array}{c} 1-\kappa_{2R},1,\kappa_{1E}\\ \kappa_{2E},0,1-\kappa_{1R} \end{array}\right). \end{array}$$

Finally, ${\mathrm{SOP}}_{L}$ is derived by plugging (32) into (30).

### 3.6. Strictly Positive Secrecy Capacity

The SPSC metric measures the probability of a guaranteed secrecy capacity of the communication system. SPSC can be mathematically calculated as [43] [Equation (17)]

(33) $$\begin{array}{c} \mathrm{SPSC}=\mathrm{Pr}\left[C_{s}\left(\gamma_{e2e},\gamma_{E}\right)>0\right]=1-{\mathrm{SOP}}_{L}{|}_{R_{s}=0}, \end{array}$$

where ${\mathrm{SOP}}_{L}$ is given in (30).

## 4. Simulation Results

In this section, we present selected numerical and Monte Carlo simulation results to verify the accuracy of the derived expressions. The values of α and β are set, similar to those adopted in [2], as α=2.296, β=2 for strong turbulence, α=4.2, β=3 for moderate turbulence, and α=8.1, β=4 for weak turbulence. Moreover, ξ=1.1 and ξ=6.7 denote strong and weak pointing error effect levels, respectively. The general FSO links’ parameters adopted for the simulations are as given in Table 1. Additionally, ω=0.10168 is considered for clear-air weather conditions and ω=0.91095 for light foggy weather conditions. Furthermore, it is assumed that $\rho_{1}=\rho_{2}=\rho$ unless otherwise stated.

**Figure 3:**
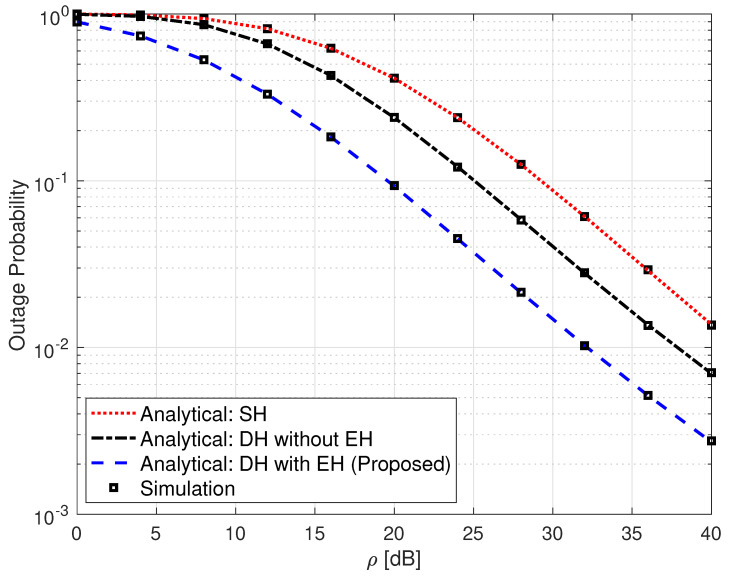
Outage probability for SH FSO, DH without EH FSO, and DH with SLIPT-based EH FSO links. $\alpha_{R}=\alpha_{D}=4.2$, $\beta_{R}=\beta_{D}=3$, $\xi_{R}=\xi_{D}=6.7$, ω=0.9065, $\gamma_{th}=1$, and τ=0.3.

Figure 3 gives a comparison of a single-hop (SH) FSO link, a non-EH DH FSO link, and the proposed SLIPT-based EH DH FSO link in terms of their OP performance metric.

In order to present a fair comparison among the systems, let us denote $P_{T}$ as the average power consumed by the communication system during *T* seconds. In this case, $P_{S}$ in (1) is given as $P_{S}=\left(2P_{T}/\right)\left(1+\tau \right)$. The analytical expressions for the SH FSO and the non-EH DH FSO links are obtained from [36] [Equation (12)] and [11] [Equation (5)], respectively. It can be seen from this figure that the proposed SLIPT-based EH DH FSO communication system outperforms the other two systems. It can also be observed from this figure that the SH FSO link presents the lowest OP performance among all considered systems, and this is due to its lack of an intermediate relay node. For instance, an OP value of $9.3\times 10^{-2}$ is obtained at ρ=20 dB for the SLIPT-based EH FSO link. To acquire the same OP value, the non-EH DH FSO link requires an extra 5 dB, whereas the SH FSO link needs approximately an extra 9 dB of ρ.

**Figure 4:**
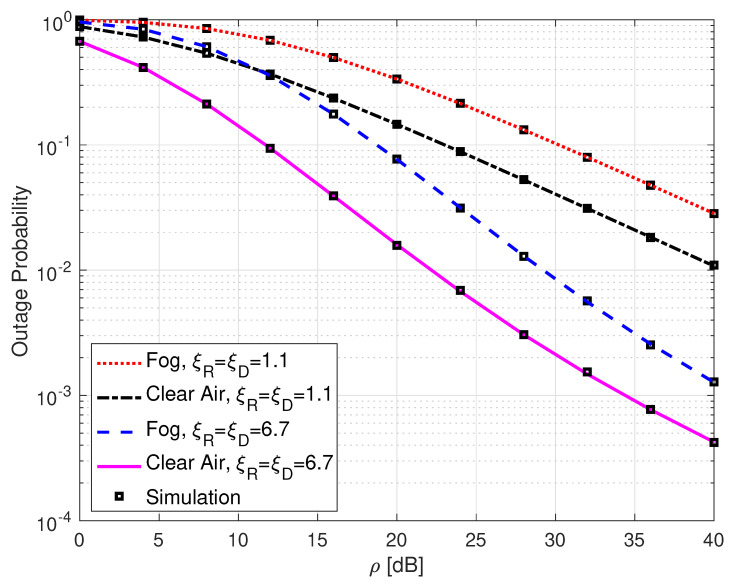
Outage probability for DH with SLIPT-based EH FSO link with different weather conditions and varying pointing error levels. $\alpha_{R}=\alpha_{D}=4.2$, $\beta_{R}=\beta_{D}=3$, $\gamma_{th}=1$, and τ=0.5.

Figure 4 presents the OP performance of SLIPT-based DH FSO links with different weather conditions and varying pointing error levels. It can be seen from the figure that the foggy weather conditions and high levels of pointing errors both deteriorate the OP performance, as expected. For example, at ρ=32 dB, a link over a clear air and with a low level of pointing errors yields an OP value of $1.5\times 10^{-3}$, while the same link but with a high level of pointing errors gives an OP value of $3.1\times 10^{-2}$.

**Figure 5:**
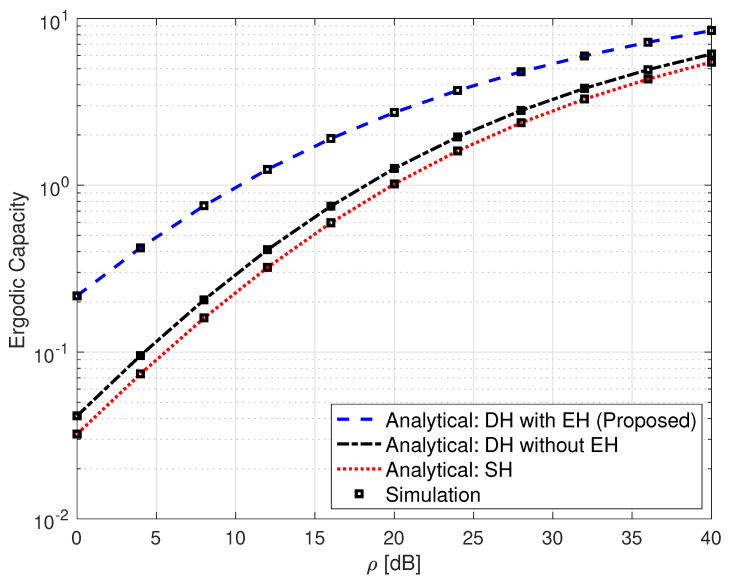
Ergodic capacity for SH FSO, DH without EH FSO, and DH with SLIPT-based EH FSO links. $\alpha_{R}=\alpha_{D}=2.296$, $\beta_{R}=\beta_{D}=2$, $\xi_{R}=\xi_{D}=6.7$, ω=0.9065, and τ=0.3.

Figure 5 exhibits a comparison of an SH FSO link, a non-EH DH FSO link, and the proposed SLIPT-based EH DH FSO link in terms of their ergodic capacity performance. The analytical expressions for the SH FSO and the non-EH DH FSO links are obtained from [36] [Equation (28)] and [11] [Equation (27)], respectively. It can be observed from this figure that the proposed SLIPT-based EH DH FSO link has a superiority in performance over the other two communication links. For instance, at ρ=32 dB, the proposed SLIPT-based EH FSO link yields an ergodic capacity of 5.97 bits/s/Hz, whereas the non-EH DH FSO and SH FSO links give ergodic capacity values of 3.811 bits/sec/Hz and 3.29 bits/s/Hz, respectively.

**Figure 6:**
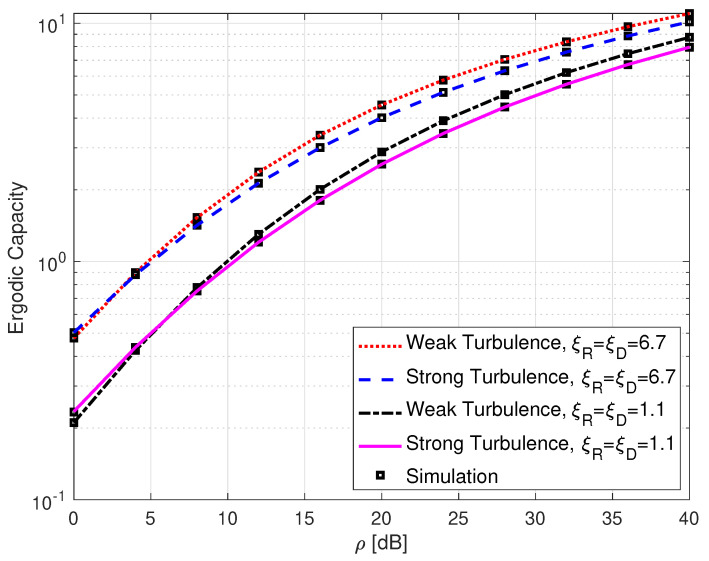
Ergodic capacity for DH with SLIPT-based EH FSO link with varying atmospheric turbulence and pointing error conditions. ω=0.10168, and τ=0.5.

Figure 6 shows the ergodic capacity performance of a SLIPT-based EH DH FSO link with different atmospheric turbulence regimes and varying pointing error levels. As expected, the erdogic capacity performance is at its best when both the atmospheric turbulence and the pointing error levels are weak.

**Figure 7:**
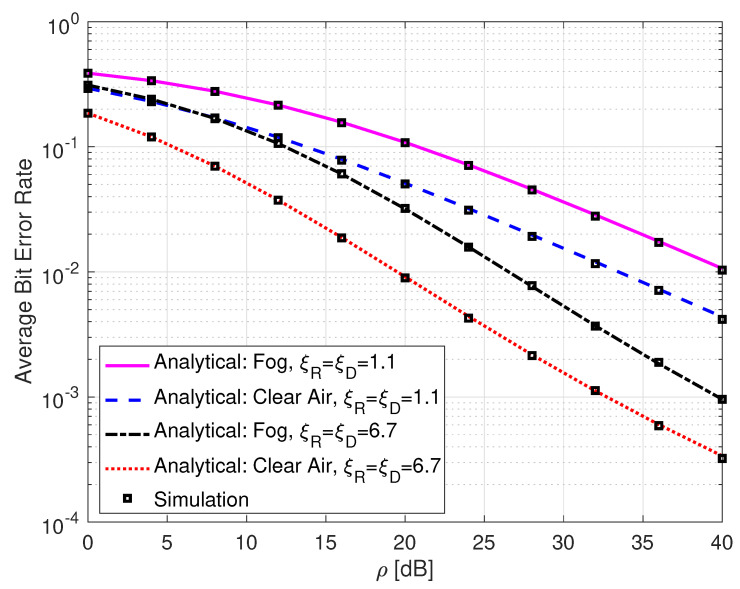
Average bit error rate for DH with SLIPT-based EH FSO link with different weather conditions and varying pointing error levels. $\alpha_{R}=\alpha_{D}=4.2$, $\beta_{R}=\beta_{D}=3$, and τ=0.5.

Figure 7 illustrates the average BER of SLIPT-based DH FSO links with different weather conditions and varying pointing error levels. It can be observed from this figure that the average BER performance is degraded by both foggy weather conditions and high pointing error levels, as expected. For instance, at ρ=28 dB, a link over a clear air and with a low level of pointing errors gives a BER value of $2.15\times 10^{-3}$, whereas the link over a foggy weather condition and with a high level of pointing errors gives a BER value of $4.51\times 10^{-2}$.

**Figure 8:**
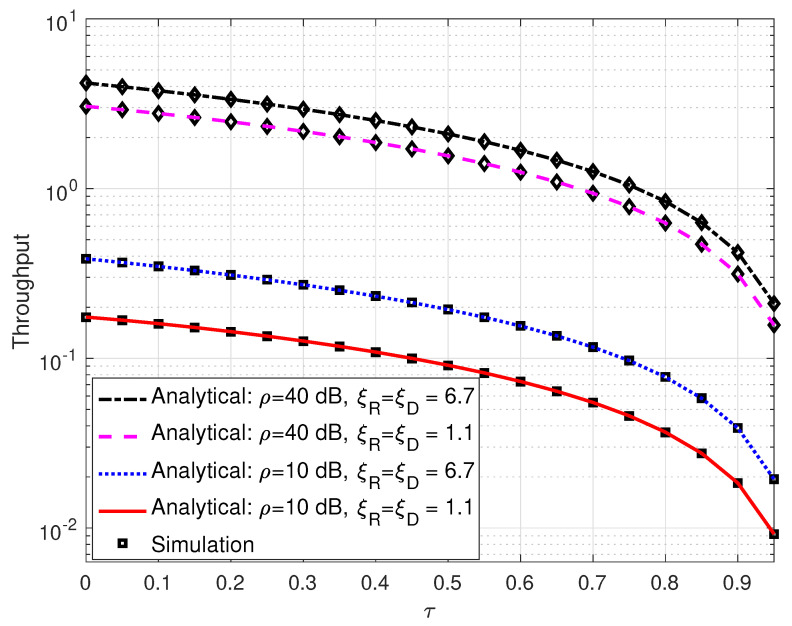
Throughput for DH with SLIPT-based EH FSO link versus τ with different levels of pointing errors and varying values of average SNR. $\alpha_{R}=\alpha_{D}=4.2$, $\beta_{R}=\beta_{D}=3$, and ω=0.9065.

Figure 8 illustrates the throughput of the proposed SLIPT-based DH FSO link versus τ, the amount of time that is dedicated for energy harvesting. This figure shows the trade-off between the amount of time for energy harvesting and the achievable throughput of the communication system. It can be observed that the throughput decreases with the increase in the amount of time for energy harvesting. Specifically, the throughput rapidly falls as τ approaches unity. For instance, a SLIPT-based DH FSO link with ρ=40 dB and ξ=6.7 experiences a 38.69% decline in throughput when τ=0.4.

**Figure 9:**
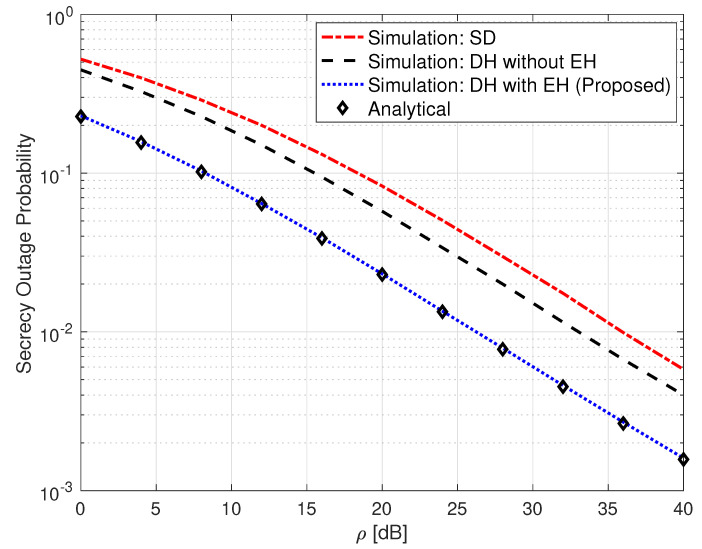
Secrecy outage probability for SH FSO, DH without EH FSO, and DH with SLIPT-based EH FSO links. $\alpha_{R}=\alpha_{D}=\alpha_{E}=2.296$, $\beta_{R}=\beta_{D}=\beta_{D}=2$, $\xi_{R}=\xi_{D}=6.7$, $\xi_{E}=1.1$, ω=0.91095, τ=0.3, and $\rho_{E}=5$ dB.

Figure 9 presents a comparison of an SH FSO link, a non-EH DH FSO link, and the proposed SLIPT-based EH DH FSO link in terms of their SOP performance metric. It can be observed from the figure that the proposed SLIPT-based FSO link outperforms the other two communication links. For instance, at ρ=28 dB, the SLIPT-based EH DH FSO link yields an SOP value of $7.8\times 10^{-3}$. However, to reach the same SOP value, the non-EH DH FSO link needs an extra 8 dB, and the SH FSO link requires approximately an extra 10 dB of ρ.

**Figure 10:**
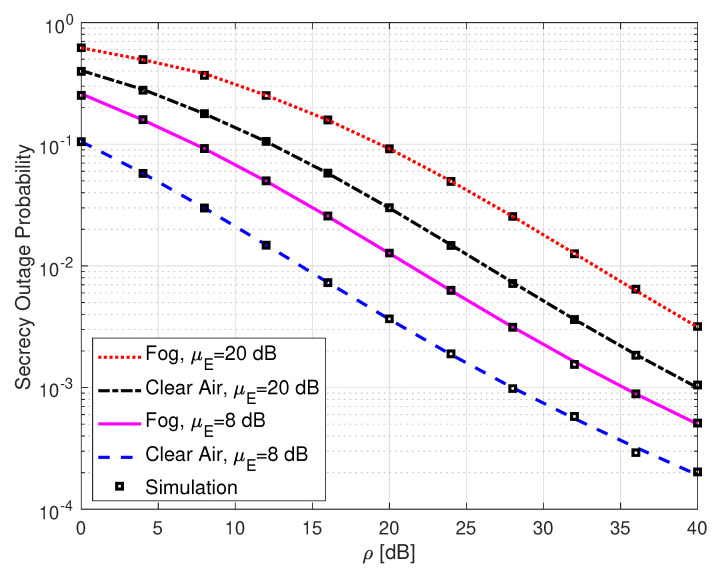
Secrecy outage probability for DH with SLIPT-based EH FSO link with different weather conditions and varying average SNR of the eavesdropper. $\alpha_{R}=\alpha_{D}=\alpha_{E}=$ 4.2, $\beta_{R}=\beta_{D}=\beta_{E}=$ 3, $\xi_{R}=\xi_{D}=$ 6.7, $\xi_{E}=$ 1.1, and τ= 0.3.

Figure 10 illustrates the SOP performance of SLIPT-based EH DH FSO links with varying average SNR of the eavesdropper and with different weather conditions. This figure demonstrates that an increase in the average SNR of the eavesdropper degrades the SOP performance. Additionally, foggy weather conditions worsen the SOP, as expected. For example, when $\mu_{E}=8$ dB and with clear air, the SOP value is $1.5\times 10^{-2}$ at ρ=12 dB. But when $\mu_{E}=20$ dB with clear air, the SOP gives a value of $1.1\times 10^{-1}$ at the same average SNR.

**Figure 11:**
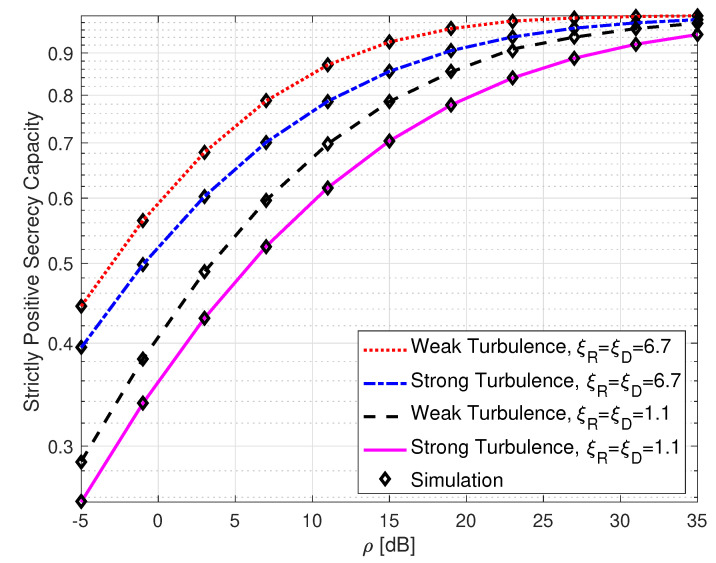
Strictly positive secrecy capacity for DH with SLIPT-based EH FSO link with varying atmospheric turbulence and pointing error conditions. $\alpha_{E}=2.296$, $\beta_{E}=2$, $\xi_{E}=1.1$, τ=0.4, and $\mu_{E}=15$ dB.

Figure 11 shows the SPSC of SLIPT-based EH DH FSO links with varying atmospheric turbulence and pointing error levels. It can be observed from the figure that the SPSC approaches unity as the value of ρ increases. It can also be seen from the figure that the SPSC has its highest value when both the atmospheric turbulence and the pointing error levels are weak. For instance, with weak atmospheric turbulence and ξ=6.7, the SPSC yields a value of $8.7\times 10^{-1}$ at ρ=11 dB. However, with strong turbulence and with ξ=1.1, the SPSC has a value of $6.2\times 10^{-1}$ at the same average SNR.

## 5. Conclusions

In this work, the performance analysis of SLIPT-based DH DF FSO communication systems is presented. Analytical expressions such as OP, ergodic capacity, average BER, throughput, SOP, and SPSC are obtained for the proposed communication system. Obtained expressions are verified with Monte Carlo simulations. Overall, it is shown that the proposed SLIPT-based FSO system outperforms the non-SLIPT FSO systems. This is due to the fact that the e2e SNR is an increasing function of the EH parameter, which in turn improves the performance of the proposed system model. It is also highlighted that there is a trade-off between the EH time and the throughput of the proposed communication system.

## Figures and Tables

**Table 1:** System’s simulation parameters.

Parameter	Value
Laser wavelength (λ)	1550 nm
FSO link distance (L)	2 km
Diameter of the APD	20 cm
Responsivity (R)	0.9
Optical-to-electrical conversion	0.9
coefficient (η)	
Thermal voltage $\left(V_{t}\right)$	25 mV
Dark saturation current $\left(I_{0}\right)$	10 nA
LOS component’s average power	1.3265
of the optical signal (Ω)	
Total scatter component’s average	2×0.1079
power of the optical signal $\left(2b_{0}\right)$	
Amount of scattering power coupled	0.596
to the LOS component (p)	
Deterministic angles	$\Phi_{A}=\pi$
	$\Phi_{B}=\pi /2$

## Data Availability

The data leading to the results presented in this study are available on request from the corresponding author.

## Abbreviations

The following abbreviations are used in this manuscript:

AC	Alternating current
AF	Amplify-and-forward
ASC	Average secrecy capacity
AWGN	Additive white Gaussian noise
BER	Bit error rate
CDF	Cumulative distribution function
DC	Direct current
DF	Decode-and-forward
DH	Dual-hop
e2e	End-to-end
EH	Energy harvesting
FSO	Free-space optical
LOS	Line-of-sight
OP	Outage probability
P-D	Photo-detector
PDF	Probability distribution function
RF	Radio frequency
RV	Random variable
SH	Single-hop
SLIPT	Simultaneous lightwave information and power transfer
SNR	Signal-to-noise ratio
SOP	Secrecy outage probability
SPSC	Strictly positive secrecy capacity

## Appendix A. Derivation of the e2e SNR’s CDF Expression

To derive the CDF expression for the e2e SNR, we rewrite $\gamma_{D}$ in (14) as $\gamma_{D}=\gamma_{D}^{\prime }\gamma_{R}$, where $\gamma_{D}^{\prime }=\mu_{0}h_{D}^{2}$, and $\mu_{0}$ is given as

(A1) $$\begin{array}{cc} \mu_{0} & =\frac{0.75V_{t}{\left(\eta_{D}R_{D}A_{D}\zeta_{D}\right)}^{2}E^{2}\left[h_{D}\right]}{\zeta I_{0}\sigma_{D}^{2}}\frac{\rho_{2}}{\rho_{1}}\left(\frac{2\tau B_{\max}^{2}}{\left(1-\tau \right)}+{\left(\frac{B_{\max}+B_{\min}}{2}\right)}^{2}\right), \end{array}$$

where $\rho_{1}=P_{S}/\sigma_{R}^{2}$ and $\rho_{2}=P_{S}/\sigma_{D}^{2}$. Now, we can rewrite (14) as

(A2) $$\begin{array}{c} F_{\gamma_{e2e}}\left(z\right)=\mathrm{Pr}\left[\min\left(\gamma_{R},\gamma_{D}^{\prime }\gamma_{R}\right)\leq z\right]. \end{array}$$

It can be noted from $\gamma_{e2e}=\min\left(\gamma_{R},\gamma_{D}^{\prime }\gamma_{R}\right)$ that $\gamma_{e2e}=\gamma_{D}^{\prime }\gamma_{R}$ when $0\leq \gamma_{D}^{\prime }<1$, and $\gamma_{e2e}=\gamma_{R}$ when $\gamma_{D}^{\prime }\geq 1$. Therefore, the CDF in (A2) can be calculated as

(A3) $$\begin{array}{c} F_{\gamma_{e2e}}\left(z\right)=\underset{I_{A1}}{\underset{︸}{\int_{0}^{1}F_{\gamma_{R}}\left(\frac{z}{c}\right)f_{\gamma_{D}^{\prime }}\left(c\right)dc}}+\underset{I_{A2}}{\underset{︸}{\int_{1}^{\infty }F_{\gamma_{R}}\left(z\right)f_{\gamma_{D}^{\prime }}\left(c\right)dc}}. \end{array}$$

The RV $\gamma_{D}^{\prime }$ follows the Malaga distribution with pointing error effect, and its PDF and CDF expressions are given as [2]

(A4) $$\begin{array}{cc} \; & f_{\gamma_{D}^{\prime }}\left(x\right)=\sum_{k=1}^{\beta_{D}}\frac{\phi_{3}}{x}G_{1,3}^{3,0}\left(\frac{\psi_{2}x^{\frac{1}{2}}}{\mu_{0}^{\frac{1}{2}}}|\begin{array}{c} \xi_{D}^{2}+1\\ \xi_{D}^{2},\alpha_{D},k \end{array}\right), \end{array}$$

(A5) $$\begin{array}{cc} \; & F_{\gamma_{D}^{\prime }}\left(x\right)=\sum_{k=1}^{\beta_{D}}\phi_{4}G_{3,7}^{6,1}\left(\frac{\psi_{2}^{2}x}{2^{4}\mu_{0}}|\begin{array}{c} 1,\kappa_{3}\\ \kappa_{4} \end{array}\right), \end{array}$$

where $\kappa_{3}=\Delta ($2$:1+\xi_{D}^{2})$, $\kappa_{4}=\Delta ($2$:\xi_{D}^{2}),\;\Delta ($2$:\alpha_{D}),\;\Delta ($2:k), $\phi_{3}=\frac{\xi_{D}^{2}A_{D}b_{k}}{4}$, $\phi_{4}=\frac{\xi_{D}^{2}A_{D}b_{k}2^{\alpha_{D}+k}}{4\pi }$, and $\psi_{2}=\frac{\xi_{D}^{2}\alpha_{D}\beta_{D}\left(g_{D}+\Omega_{D}^{\prime }\right)}{\left(g_{D}\beta_{D}+\Omega_{D}^{\prime }\right)\left(1+\xi_{D}^{2}\right)}$. $A_{i}$ and $b_{k}$ are given in (6).

To solve the integral in (A3), we write the PDF of $\gamma_{D}^{\prime }$ in (A4) in terms of power series, by employing [44] [Equation (9.303)] and [40] [Equation (07.31.02.0001.01)], as

(A6) $$\begin{array}{cc} f_{\gamma_{D}^{\prime }}\left(x\right) & =\sum_{k=1}^{\beta_{D}}\frac{\Phi_{1}x^{\frac{\xi_{D}^{2}}{2}-1}}{\mu_{0}^{\frac{\xi_{D}^{2}}{2}}}+\sum_{k=1}^{\beta_{D}}\sum_{n=0}^{\infty }\frac{\Phi_{2}x^{\frac{n+\alpha_{D}}{2}-1}}{\mu_{0}^{\frac{n+\alpha_{D}}{2}}}+\sum_{k=1}^{\beta_{D}}\sum_{n=0}^{\infty }\frac{\Phi_{3}x^{\frac{n+k}{2}-1}}{\mu_{0}^{\frac{n+k}{2}}}, \end{array}$$

where

(A7) $$\begin{array}{cc} \; & \Phi_{1}=\phi_{3}\Gamma \left[\alpha_{D}-\xi_{D}^{2}\right]\Gamma \left[k-\xi_{D}^{2}\right]\psi_{2}^{\xi_{D}^{2}};\\ \; & \Phi_{2}=\frac{\phi_{3}\Gamma \left[\xi_{D}^{2}-\alpha_{D}\right]\Gamma \left[k-\alpha_{D}\right]{\left(\alpha_{D}-\xi_{D}^{2}\right)}_{n}\psi_{2}^{n+\alpha_{D}}}{n!\Gamma \left[1+\xi_{D}^{2}-\alpha_{D}\right]{\left(1+\alpha_{D}-\xi_{D}^{2}\right)}_{n}{\left(1+\alpha_{D}-k\right)}_{n}};\\ \; & \Phi_{3}=\frac{\phi_{3}\Gamma \left[\xi_{D}^{2}-k\right]\Gamma \left[\alpha_{D}-k\right]{\left(k-\xi_{D}^{2}\right)}_{n}\psi_{2}^{n+k}}{n!\Gamma \left[1+\xi_{D}^{2}-k\right]{\left(1+k-\xi_{D}^{2}\right)}_{n}{\left(1+k-\alpha_{D}\right)}_{n}}. \end{array}$$

In (A7), ${\left(a\right)}_{n}$ represents the Pochhammer symbol [40] [Equation (06.10.02.0001.01)]. Now, the integral $I_{A1}$ in (A3) can be rewritten by using (12) and (A6) in $I_{A1}$, and with the help of [40] [Equation (07.34.17.0012.01)], as

(A8) $$\begin{array}{cc} I_{A1} & =\sum_{k=1}^{\beta_{R}}\sum_{l=1}^{\beta_{D}}\frac{\Phi_{1}\phi_{2R}}{\mu_{0}^{\varrho_{1}}}\int_{0}^{1}c^{\varrho_{1}-1}G_{7,3}^{1,6}\left(\frac{2^{4}\mu_{R}c}{\psi_{1R}^{2}z}|\begin{array}{c} 1-\kappa_{2R},1\\ 0,1-\kappa_{1R} \end{array}\right)dc+\sum_{k=1}^{\beta_{R}}\sum_{l=1}^{\beta_{D}}\sum_{n=0}^{\infty }\frac{\Phi_{2}\phi_{2R}}{\mu_{0}^{\varrho_{2}}}\int_{0}^{1}c^{\varrho_{2}-1}G_{7,3}^{1,6}\left(\frac{2^{4}\mu_{R}c}{\psi_{1R}^{2}z}|\begin{array}{c} 1-\kappa_{2R},1\\ 0,1-\kappa_{1R} \end{array}\right)dc\\ \; & +\sum_{k=1}^{\beta_{R}}\sum_{l=1}^{\beta_{D}}\sum_{n=0}^{\infty }\frac{\Phi_{3}\phi_{2R}}{\mu_{0}^{\varrho_{3}}}\int_{0}^{1}c^{\varrho_{3}-1}G_{7,3}^{1,6}\left(\frac{2^{4}\mu_{R}c}{\psi_{1R}^{2}z}|\begin{array}{c} 1-\kappa_{2R},1\\ 0,1-\kappa_{1R} \end{array}\right)dc, \end{array}$$

where $\varrho_{1}=\frac{\xi_{D}^{2}}{2}$, $\varrho_{2}=\frac{n+\alpha_{D}}{2}$, and $\varrho_{3}=\frac{n+l}{2}$. The closed-form solution for $I_{A1}$ can be obtained with the aid of [40] [Equation (07.34.21.0084.01)], and after some simplification by using [40] [Equation (07.34.17.0012.01)], $I_{A1}$ in (A8) is given as

(A9) $$\begin{array}{cc} I_{A1} & =\sum_{k=1}^{\beta_{R}}\sum_{l=1}^{\beta_{D}}\frac{\Phi_{1}\phi_{2R}}{\mu_{0}^{\varrho_{1}}}G_{4,8}^{7,1}\left(\frac{\psi_{1R}^{2}z}{2^{4}\mu_{R}}|\begin{array}{c} 1,\kappa_{1R},1+\varrho_{1}\\ \varrho_{1},\kappa_{2R},0 \end{array}\right)+\sum_{k=1}^{\beta_{R}}\sum_{l=1}^{\beta_{D}}\sum_{n=0}^{\infty }\frac{\Phi_{2}\phi_{2R}}{\mu_{0}^{\varrho_{2}}}G_{4,8}^{7,1}\left(\frac{\psi_{1R}^{2}z}{2^{4}\mu_{R}}|\begin{array}{c} 1,\kappa_{1R},1+\varrho_{2}\\ \varrho_{2},\kappa_{2R},0 \end{array}\right)\\ \; & +\sum_{k=1}^{\beta_{R}}\sum_{l=1}^{\beta_{D}}\sum_{n=0}^{\infty }\frac{\Phi_{3}\phi_{2R}}{\mu_{0}^{\varrho_{3}}}G_{4,8}^{7,1}\left(\frac{\psi_{1R}^{2}z}{2^{4}\mu_{R}}|\begin{array}{c} 1,\kappa_{1R},1+\varrho_{3}\\ \varrho_{3},\kappa_{2R},0 \end{array}\right). \end{array}$$

Furthermore, $I_{A2}$ in (A3) can be solved as

(A10) $$\begin{array}{c} I_{A2}=F_{\gamma_{R}}\left(z\right)\int_{1}^{\infty }f_{\gamma_{D}^{\prime }}\left(c\right)dc=\left(1-F_{\gamma_{D}^{\prime }}\left(1\right)\right)F_{\gamma_{R}}\left(z\right), \end{array}$$

where $F_{\gamma_{D}^{\prime }}\left(1\right)$ is given in (A5) and $F_{\gamma_{R}}\left(z\right)$ can be obtained from (12). Finally, the CDF of e2e instantaneous SNR is derived by plugging (A9) and (A10) into (A3) as given in (15). Please note that, when performing numerical calculations, the upper-limit of the two infinite summations in (A9) are truncated to finite *N* terms in a way that is minimizing the convergence error. One can choose a suitable value of *N* that yields a negligible convergence error. We consider N=15 for all the analytical curves presented in Section 4.
